# The Bioprotective Effects of Polyphenols on Metabolic Syndrome against Oxidative Stress: Evidences and Perspectives

**DOI:** 10.1155/2019/6713194

**Published:** 2019-11-30

**Authors:** Kui Liu, Miao Luo, Shuang Wei

**Affiliations:** Department of Respiratory and Critical Care Medicine, Key Laboratory of Pulmonary Diseases of Health Ministry, Tongji Hospital, Tongji Medical College Huazhong University of Science and Technology, Wuhan 430030, China

## Abstract

Polyphenols are the general designation of various kinds of phytochemicals, mainly classified as flavonoids and nonflavonoids. Polyphenolic compounds have been confirmed to exhibit numerous bioactivities and potential health benefits both *in vivo* and *in vitro*. Dietary polyphenols have been shown to significantly alleviate several manifestations of metabolic syndrome, namely, central obesity, hypertension, dyslipidemia, and high blood sugar. This review is aimed at discussing the bioprotective effects and related molecular mechanisms of polyphenols, mainly by increasing antioxidant capacity or oxygen scavenging capacity. Polyphenols can exert their antioxidative activity by balancing the organic oxidoreductase enzyme system, regulating antioxidant responsive signaling pathways, and restoring mitochondrial function. These data are helpful for providing new insights into the potential biological effects of polyphenolic compounds and the development of future antioxidant therapeutics.

## 1. Introduction

**Figure 1:**
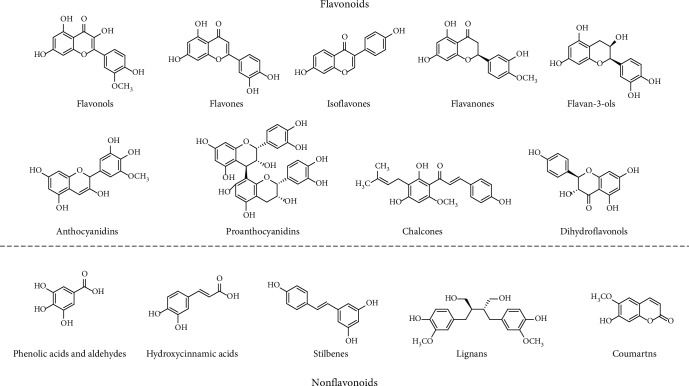
Representative chemical structures of major groups of polyphenols, generally classified as flavonoids and nonflavonoids.

Polyphenols are compounds derived from plants, which exert protective effects against various environmental stresses in plants [1]. Polyphenols represent the largest group of phytochemicals that possess aromatic rings and ligand groups (mainly hydroxyl combined with different carbon sites). Natural polyphenols are secondary metabolites, and more than ten thousand natural polyphenols have been identified and evaluated thus far [2]. Depending on their structural features, polyphenols can be classified into flavonoids and nonflavonoids [3]. Flavonoids share a common structure of a 15-carbon skeleton, composed of two aromatic rings, connected through a heterogeneous pyrone C ring. This carbon bridge structure can be denoted as C6-C3-C6. As heterocyclic ring-linked groups, flavonoids harbor several subgroups, namely, flavonols, flavones, isoflavones, flavanones, anthocyanidins, flavan-3-ols, and minor subclasses of flavonoids, such as chalcones [3]. In contrast, nonflavonoids refer to phenolic acids, hydroxycinnamic acids, stilbenes, lignans, and coumarins [4, 5]. Representative chemical structures of flavonoids and nonflavonoids are shown in Figure 1. The classifications, compositions, and sources of polyphenols are shown in Table 1.

Although polyphenols do not belong to the category of essential nutrients, ingestion of a certain amount of polyphenolic compounds shows a positive influence on body growth and suppresses the occurrence and development of diseases. Several *in vivo* and *in vitro* studies have reported that polyphenolic compounds exhibit multiple bioactivities and potential health benefits, such as protection of cardiovascular diseases, disruption of oxidation and inflammatory response, growth inhibition of tumor cells and bacteria, and retardation of the aging process [2]. Metabolic syndrome (MetS) is a common clinical syndrome manifested as several abnormal medical phenotypes, such as central obesity, hypertension, dyslipidemia, as well as high blood sugar. MetS is a major trigger for the occurrence and progression of type II diabetes (T2D) and cardiovascular disease. With great advances in basic redox biology, the most important pathophysiology of MetS is considered to be abnormal systemic oxidative stress (OS) via increased production of free radicals and redox imbalance. Increased OS in the body triggers several diseases, such as diabetes [6], cardiovascular diseases like atherosclerosis [7], Alzheimer's disease [8], and even cancer [9]. OS is closely related to systemic inflammation, endothelial dysfunction, metabolic abnormality, and DNA damage [10]. Nowadays, the preferred management of MetS involves maintenance on a regular and standard diet with increased intake of fruits and vegetables, with reducing the consumption of high-fat, high-salt, and high-sugar substances and optimizing the diet structure, improving physical exercise training, and reducing alcohol consumption [11]. Therefore, a new preventive solution is urgently needed to reduce the morbidity and development of MetS. Until now, several studies have confirmed that supplementation with single/multiple polyphenolic compounds or specific phenolic extracts exhibits protective effects on MetS in cell and animal models. Tian et al. indicated that polyphenols that are extracted from green tea (GT) cut down fat deposition in high fat-fed rats by inhibition of extracellular signal-regulated kinase (ERK) 1/2-peroxisome proliferator-activated receptor (PPAR) *γ*-adiponectin pathway [12]. They also reported that intake of green tea polyphenols (GTP) reduces the prevalence of coronary heart disease in the middle-aged and elderly Chinese populations to some extent [13]. Resveratrol (RSV, a form of nonflavonoid) treatment was demonstrated to attenuate the hyperpermeability and the overexpression of cav-1 induced by high glucose in a dose-dependent manner. RSV was confirmed to downregulate the increased expressions of vascular endothelial growth factor (VEGF) and kinase insert domain receptor (KDR or VEGF receptor-2) induced by high glucose [14]. In a randomized, controlled, double-blinded clinical trial, Faghihzadeh et al. found that RSV supplementation for 12 weeks significantly decreased the level of alanine aminotransferase (ALT) and arrested hepatic steatosis in nonalcoholic fatty liver disease (NAFLD) patients compared to the control group (*P* < 0.05) [15]. Therefore, polyphenols, as powerful antioxidants commonly found in fruits and vegetables, are considered to be a potential therapeutic candidate for MetS [16].

The purpose of this review is to renew and supplement information about the effects of polyphenols on MetS and related antioxidative mechanisms, derived from cell studies, animal studies, and especially, interventional human studies.

## 2. The Potential Roles of Polyphenols in MetS

Polyphenols are biomolecules derived from plant origin and have been proven to exhibit antioxidant and anti-inflammatory activities. Several lines of evidence have suggested that polyphenols, at certain doses, might delay or prevent MetS onset by decreasing body weight, blood pressure (BP), and blood glucose, and by improving abnormal lipid metabolism [17].

### 2.1. Hyperglycemia and Diabetes Mellitus

The International Diabetes Federation (IDF) has predicted that there were 451 million diabetes patients globally in 2017, increasing with an alarming rate, and by 2045, diabetes patients are estimated to be drastically increased up to 693 million. Majority of those patients were classified as type 2 diabetes (T2D) patients [18]. The main clinical characteristics of T2D involve systematic insulin resistance and relative insufficiency of insulin secretion [19].

In a clinical trial, 50 participants exhibiting more than one abnormal medical phenotypes of MetS were recruited and randomly administered 8 g dried grape pomace (containing mainly catechins and proanthocyanidins) or placebo daily. After six weeks of supplementation with grape pomace, basal insulin was decreased from to 8.5 to 5.5 *μ*U/mL, showing a significant improvement in fasting insulinemia. No significant change was found in both fasting and postprandial glucose between the pre- and postintervention period. Moreover, homeostatic model assessment-insulin resistance (HOMA-IR) was decreased from 2.1 to 1.4 while quantitative insulin sensitivity check index (QUICKI) was increased from 0.35 to 0.42. Previous clinical studies have revealed that anthocyanins and RSV prevent incidence of diabetes, by reducing the levels of blood glucose and hemoglobin A1c (HbA1c), promoting secretion efficiency of pancreatic *β* cells, and ameliorating systematic insulin resistance [20]. In another randomized, controlled, crossover study, 12 healthy men participated in interventions with either 1.562 g gallic acid equivalent (GAE) beverage or a placebo beverage without polyphenols (CD) randomly, followed by a standard meal after 3 hours. Compared to the CD group, a 36% increase of insulin sensitivity index (SI) was found in the GAE group, while a 31% and 18% decrease was observed in the postprandial insulin incremental area (iAUC0-5h) and insulin secretion index, respectively, after polyphenol supplementation. Among the phenolic metabolites, gallic acid was positively related to the SI index (*r* = 0.588) but negatively with insulin secretion and response (*r* = −0.604) [21]. Polyphenol, added to the usual diet, could contribute to improve blood sugar metabolism but could have different influences in different individuals. In obese participants, with supplementation of mangoes that are rich in gallotannin (polymeric form of gallic acid) for 6 weeks, HbA1c was decreased by 18% [22]. Rienks et al. have systematically analyzed eight studies and the relationship between polyphenols and T2D by using a meta-analysis. In this analysis, most flavonoids, especially flavanones and anthocyanidins, exhibited nonlinear dose-response correlations with T2D. However, phenolic acids, belonging to nonflavonoids, showed a linear relationship. This difference might be attributed to the difference in their structures. The researchers concluded that additional dietary polyphenols, especially flavonoids, might exert an important influence on the improvement of T2D [23].

This section summarizes the results from various clinical trials of polyphenolic compounds as antidiabetic agents. It has been identified that natural polyphenols show antidiabetic effects, by reducing the levels of blood glucose and HbA1c, increasing the secretion efficiency of pancreatic *β* cells, and ameliorating systematic insulin resistance. Dietary supplements with polyphenols have been confirmed as an indispensable part of diabetes diet therapy in clinical trials. However, how diabetes alters the bioavailability and bioactivity of polyphenols is not well understood. It might contribute to an increase in their inherent effects and clinical consequences.

### 2.2. Obesity

In the past few decades, the global incidence rate of obesity has dramatically increased. Epidemiological survey results show that, from 1975 to 2016, the incidence rate of obesity (defined as body mass index (BMI) ≥ 25 kg/m^2^) was raised by affecting 40% more adults (21% in men and 24% in women). The morbidity rate of obesity (BMI ≥ 30 kg/m^2^) in men is growing faster than that in women, with the rate increasing for men from 3% to 12% and for women only doubling more than twice (from 7% to 16%) [24]. It has been predicted that more than 1 billion people will be obese by 2030 [25].

Marranzano et al. designed a cohort trial to elucidate the relationship between obesity and estimated dietary flavonoid intake. Intake of flavonoids was negatively correlated with BMI ≥ 25 and the odds ratio (OR) was about 66%. Surprisingly, among the subclasses of flavonoids, only flavanones showed this similar correlation (OR = 0.68) after adjustment for confounding factors. These data indicated that higher intake of flavonoid resulted in the loss of body weight [26]. In a controlled and double-blind study, 95 overweight individuals were randomly administered 900 mg of a citrus-based polyphenol (mainly catechin and naringin) extract or a placebo taken daily with meals. Compared with the placebo group, waist and abdominal fat and hip circumference in the intervention arm were significantly decreased (waist -5.71% vs. -1.56%, hip -4.71% vs. -1.35%, and fat -9.73% vs. -3.18%) compared to those in the control arm. Furthermore, OS was also lowered by increasing superoxide dismutase (SOD) and glutathione (GSH) and reducing serum malondialdehyde (MDA) levels [27]. Consistent with other clinical trials, 33 participants were recruited with either BMI of more than 25 kg/m^2^ or a waist circumference beyond 94 cm in men or 80 cm in women. They were randomly distributed into two groups that were administered a diet supplemented either with 500 mg C. fimbriata extract (gallic acid) or placebo. Waist to hip ratio (WHR) were measured to be decreased by 3% in the intervention group, compared to 1% in the control (*P* < 0.05). In addition, body weight, hip circumference, blood pressure, triglyceride levels, and fat accumulation were all found to be markedly decreased in the polyphenol-intake group compared to the placebo group (*P* < 0.05). These results indicated that the extract of C. fimbriata combined with diet control and adequate exercise might significantly reduce central obesity, one of the vital phenotypes of MetS [28].

Of course, there were also some studies that reported inconsistent or controversial results. The accretion of green tea (GT, mainly flavan-3-ols) for 12 weeks in sixty subjects (BMI > 18 kg/m^2^) did not exert a significant influence on body weight, BMI, fat deposition, and resting energy expenditure [29]. Another 12-month randomized, double-blind, placebo-controlled clinical trial recruited 937 postmenopausal females (BMI ≥ 25.0 kg/m^2^). These individuals were randomized into different groups, the GT extract group, containing individuals administered 843 mg (-)-epigallocatechin-3-gallate, and the remaining in the placebo group. The changes in BMI, overall fat content, percentage of body fat, or bone mineral density in 12 months were not statistically different [30].

In conclusion, this section reviewed the advances of the benefit of polyphenol supplementation in clinical trials. Consumption of polyphenols has been proposed to result in favorable improvement in fat deposition and extenuates inflammation, abnormal blood glucose metabolism, and oxidative unbalance status in overweight subjects, although some negative results were reported. In addition, polyphenol supplementation was well tolerated with no adverse effect [31–34]. Therefore, future researches are warranted to confirm these results over a longer period and discover the potential mechanisms of action of the polyphenols.

### 2.3. Hypertension

Elevated BP in MetS is a predominant risk factor contributing to cardiovascular damage. With an increase in blood pressure, multiple target organ dysfunctions can occur. Many studies have found that different polyphenols have different degrees of effects on BP.

Daily intake of 25 g chocolate, containing high content of polyphenols, resulted in significant decrease of about 5.93 mmHg and 6.4 mmHg in systolic and diastolic BP, respectively, after administration for 8 weeks [35]. Taubert et al. confirmed that even low habitual 6.3 g dark chocolate intake (equivalent with 30 mg polyphenols) can have a favorable clinical effect on individuals with untreated prehypertension or stage 1 hypertension not accompanied with targeted organ damage risks. From the baseline to end of trial for 18 weeks, mean diastolic BP dropped by 1.9 mmHg and systolic BP declined more prominently by 2.9 mmHg accompanied by a decreasing trend in hypertension prevalence from 86% to 68% in the experimental group. Moreover, the decline in BP was associated with a progressive elevation of S-nitrosoglutathione concentration at about 0.23 nmol/L, indicating that OS played an important role in this process [36].

It should be noted that not all polyphenols have the same effect on systolic and diastolic BP. A systemic review and meta-analysis of twenty-four randomized-controlled clinical trials (RCTs) recruited 1106 participants to explore the efficacy of flavonoid-rich cocoa (FRC) on alleviating potential cardiovascular damage and to evaluate their possible correlation. When administered with FRC for up to 2 weeks, systolic BP dropped by 1.63 mmHg (95%CI = 0.13, 3.12; *P* = 0.033), while diastolic BP exhibited no changes [37]. Nevertheless, a randomized, double-blind crossover study showed the purveyance of orange juice, abundant in various kinds of polyphenols (mainly anthocyanins), reduced both systolic and diastolic BP. This intervention decreased systolic BP from 128 ± 1 mmHg to 124 ± 2 mmHg (*P* < 0.05) and decreased diastolic BP from 79 ± 1 mmHg to 76 ± 1 mmHg (*P* < 0.05) [38]. Nevertheless, some studies have reported controversial results. Koli et al. evaluated the potential functions of regular ingestion of flavonoid-abundant dark chocolate on BP, while restricting snack intake intervention, for 8 weeks. Intake of dark chocolate exhibited no significant benefits on 24 h resting BP (142 ± 11.5/89 ± 8.4 mmHg in initial vs. 142 ± 14.2/88 ± 9.4 mmHg later, *P* > 0.05). The authors concluded that dark chocolate supplement showed no significant changes in BP or other cardiovascular risk factors during a reduced snack period [1]. As for nonflavonoids, in a randomized-controlled clinical trial, 37 middle-aged and older participants were enrolled and were either provided with 480 mL of tart cherry beverage (high in gallic acid) or control drink (only energy and sugar content) daily for 12 weeks. At the beginning, individuals with high polyphenols retained superior mean systolic BP than the control group (141.4 mmHg vs. 133.4 mmHg). At the end of this study, individuals consuming high polyphenolic compounds showed lower systolic BP than the control group (137.3 mmHg in the test group vs. 138.8 mmHg in the control group). However, no statistically significant change in diastolic BP was observed in either treated or control group [39]. Fang et al. conducted a clinical study showing that mango (full of gallotannin-derived polyphenols) supplementation for 6 weeks significantly decreased systolic BP (but not diastolic BP) in the individuals; however, no expected change in BP occurred in obese individuals [22].

Taken together, daily polyphenol-rich food intake lowers the blood pressure in adults, and it is a plausible intervention for improving cardiovascular health. However, some clinical studies have shown inconsistent results. Therefore, further clinical trials are urgently needed in a larger population and for longer durations to further elucidate the protective role of polyphenols in hypertension.

### 2.4. Dyslipidemia

There is no doubt that hyperlipidemia is one of the major risk factors for cardiovascular disease. In the USA, about 45.0% and 32.0% of females were found to have increased levels of total cholesterol (TC) and low-density lipoprotein cholesterol (LDLC), respectively [40].

Erlund et al. showed that, compared to the control, serum high-density lipoprotein cholesterol (HDLC) level significantly increased in the berry-eating group (abundant flavan-3-ols, flavonols, and flavanones) compared to the control group (5.2% vs. 0.6%, *P* < 0.05), while TC and triacylglycerol (TG) levels remained unchanged [41]. Another RCT indicated that green tea extract consumption in individuals for 1 year led to a marked decrease in circulating TC (-2.1% vs. 0.7%; *P* < 0.01), LDLC (-4.1% vs. 0.9%; *P* < 0.0001), and non-high-density lipoprotein cholesterol (-3.1% vs. 0.4%; *P* < 0.01) compared with placebo. The dose of serum HDLC remained unchanged, whereas TG concentration increased to 3.6% in the experimental group compared to the control group (3.6% vs. -2.5%, *P* < 0.05) [42]. Another clinical study showed that intake of 122 mg Goishi tea polyphenols daily increased HDLC level and decreased TC level in the individuals, exhibiting the potential beneficial effect of reducing risks of arteriosclerosis and cardiovascular diseases [43]. A systematic review including 17 clinical trials (*n* = 1356) concluded that intake of green tea epigallocatechin gallate (EGCG) from 107 to 856 mg/d apparently decreased LDLC by 9.29 mg/dL (95% CI, -12.27 to -6.31), and this protective effect mainly depends on the lipid level at baseline [44]. With consumption of 500 mg aronia extract, containing anthocyanins, proanthocyanidins, and hydroxycinnamic acids, the aronia-interventional group presented a favorable effect in microlipid metabolism. After 12 weeks of treatment, in the peripheral circulating bloodstream, TC concentration dropped by 8%, while LDLC level was increased up to 11%, compared to the placebo. Even LDL receptor protein in mononuclear cells was detected to be reduced by 56% from the baseline, whereas the placebo group showed no significant changes [45].

In conclusion, favorable changes were revealed in TC, LDLC, and HDLC levels after the administration of polyphenols in several clinical trials. These findings can guide us to suggest people to consume more fruits and vegetables containing abundant polyphenols that might improve blood lipid profiles, and thereby, prevent dyslipidemia. More studies are needed to further identify the favorable effects of these polyphenolic compounds on dyslipidemia and explore their underlying molecular mechanisms.

### 2.5. Nonalcoholic Fatty Liver Disease

Nonalcoholic fatty liver disease (NAFLD) is the most common chronic liver disease. NAFLD is closely related to the abnormal metabolism of carbohydrates and lipids and triggers insulin resistance and T2D. The favorable effects of polyphenols on NAFLD have been investigated in several clinical trials.

The supplementation of 500 mg RSV for 12 weeks significantly decreased the alanine aminotransferase (ALT) concentration and hepatic steatosis in 50 NAFLD patients compared to the placebo (*P* < 0.05); however, no expected improvements in anthropometric measurements, insulin resistance (IR), lipid profile, and BP were observed (*P* > 0.05) [15]. This group also found that RSV (at the same concentration for the same period) consumption combined with lifestyle change exhibited superior effects compared to lifestyle modification alone [46]. The reduction of AST, plasma glucose, LDLC, TC, insulin resistance index, and TNF-*α* was also observed in NAFLD patients after administration of edible RSV capsules [47]. A short-term (8-week) daily intake of curcumin also extenuated liver fat and serum levels of ALT and AST in patients diagnosed with NAFLD [32]. Daily consumption of 70 mg of curcumin was found to alleviate liver fat content by 78.9% in NAFLD patients, while only 27.5% improvement was observed in placebo group patients. Most physiological and biochemical features of NAFLD were improved, including levels of TC, LDLC, ALT, and AST. During the trial, curcumin was found to be safe and well tolerated in the patients [48].

In summary, increasing evidence has shown the satisfactory effectiveness of polyphenolic compounds in NAFLD. In addition, it has been shown that polyphenol (flavonoids and nonflavonoids) supplements combined with lifestyle changes ameliorated both occurrence and progression of NAFLD, which exhibited a more favorable effect than lifestyle changes alone. Therefore, polyphenol supplement plus healthy lifestyle might be a feasible choice for patients with NAFLD.

## 3. Antioxidant Effects of Polyphenols on MetS

**Figure 2:**
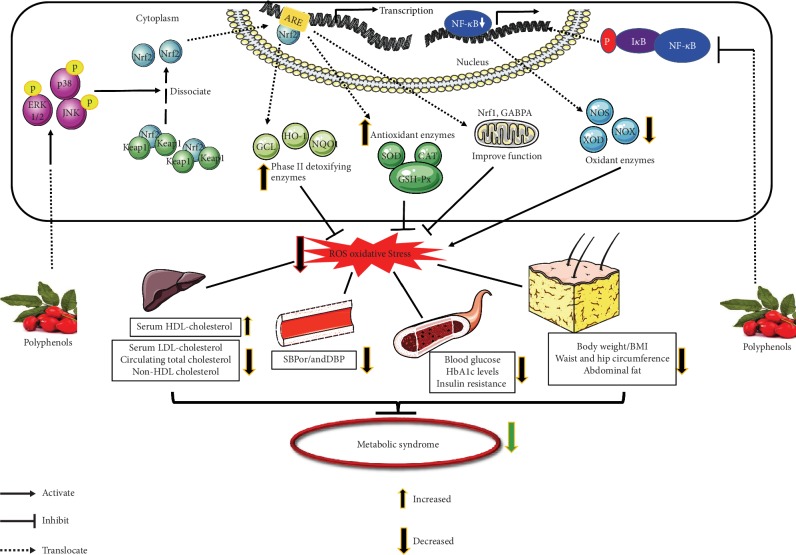
Schematic representation of the main antioxidative mechanisms responsible for MetS regulated by polyphenols.

The detailed mechanism underlying the protective effects of polyphenol is not yet understood; however, oxidative stress, as one of the vital pathogeneses of MetS, suggests many targeted therapies for these diseases. For the purpose of establishing a clearer and deeper understanding of the roles of various kinds of polyphenols in MetS, we summarized the results of several *in vitro* and *in vivo* studies on antioxidant effects and related mechanisms in different models, as shown in Table 2 [49–64]. We also summarized the results of studies on the protective effects of polyphenols against MetS in clinical trials (as shown in Table 3) [15, 21, 22, 27, 28, 36, 38, 39, 41, 42, 46, 47, 65–70].  Figure 2 summarizes the schematic representation of the principal antioxidative mechanisms of polyphenols in various cellular models.

### 3.1. Antioxidant Properties of Polyphenols

The antioxidant properties of polyphenols and secondary metabolites depend on the chemical construction of attached functional groups, more specifically, the permutation of functional groups about the nuclear structure. The number of hydroxyl residues greatly affects antioxidant activity by scavenging radicals and disrupting metal ion chelation [71]. In addition, other structures like O-methylation, 2–3 double bond, and 4-oxo, carbohydrate moieties, and degree of polymerization play roles in antioxidant activity [71]. Antioxidant activities of polyphenols are associated with their capacity to eliminate high levels of reactive oxygen species (ROS).

Bai et al. performed a study to investigate the biological characteristics of polyphenolic extracts from apple pomace using antioxidant assays and high-performance liquid chromatography (HPLC) analysis. The study identified that the antioxidant ability among apple polyphenolic extracts was highest in procyanidins B2, followed by chlorogenic acid, hyperin, quercetin, caffeic acid, syringin, cinnamic acid, and phloridzin [72]. Another study showed that O-methylation of the hydroxyls of the catechol B ring resulted in a decrease of the antioxidant activity of the parent compounds. Antioxidant activity from strong to weak is as follows: quercetin>epicatechin>catechin. In addition, pH value also affected antioxidant activity of polyphenols in this assay; lower antioxidant activity was observed at acidic pH. It suggested that the same polyphenols might exhibit different antioxidant capacities in different parts of the body and also provided a possible strategy to enhance the efficacy of dietary polyphenolic compounds [73]. Lemańska et al. reported that O-methylation modification of the quercetin and luteolin undermined the radical scavenging capability by affecting the donating property of electron and hydrogen atom. This study also provided a new insight into the mechanisms of catechol O-methylation on radical scavenging [74].

Cellular antioxidant activity (CAA) assay, combined with *in vitro* digestion, is notably more effective in detecting antioxidant activity than a simple chemical assay alone [75]. Sun et al. reported that flavonoids and phenolic acids in fresh citrus fruits significantly increased CAA values in a human intestinal HepG2 cell model [56]. In *ex vivo* studies, the order of ability to donate electron and antioxidant activity was gallic acid>quercetin>rutin>vanillin>acetylsalicylic acid. These compounds were considered to play roles in the antioxidant properties of *S*. *aegyptiaca* [57].

Increasing evidence has shown that polyphenols have strong antioxidant properties. Currently, it has been speculated that the antioxidant efficiency of polyphenols suppressed ROS generation by means of inhibiting the catalytic activity of related synthetic enzymes, scavenging of ROS, and upregulating the protective antioxidant defensive network [76, 77].

### 3.2. Antioxidant Signal Pathway Regulation

The antioxidant response elements (AREs), namely, cis-acting regulatory elements, participate in regulating the expression of special gene encoding enzymes involved in antioxidant and phase II detoxifying reactions [78]. The nuclear transcription factor, erythroid 2-like 2 (Nrf2), a central conditioner of AREs, is commonly associated with the Kelch-like ECH-associated protein 1 (Keap1) in the cytoplasm, and subsequently, degraded by the proteasome. However, in the stimulation of oxidative stress, Keap1 cysteine residues were oxidized, which led to Nrf2 dissociation and translocation into the nucleus, where it interacted with AREs, and thus, upregulated expression of downstream genes [79].

Polyphenols, as powerful antioxidants, were confirmed to promote the Nrf2 nuclear translocation under oxidative stress. On one hand, polyphenols protected against tert-Butyl hydroperoxide- (t-BHP-) induced oxidative injury via upregulation of the expression of primary and phase II detoxifying genes, especially enzyme-encoding genes. Heme oxygenase-1 (HO-1), genetically encoded by *Hmox-1*, is a rate-limiting enzyme of heme catabolism, which gets transformed into carbon monoxide (CO), biliverdin, and ferrous iron. The interaction of biliverdin and bilirubin constituted an effective antioxidant, namely, biliverdin reductase (BVR), to reduce OS [80]. The polyphenol-rich extract of the *Nymphaea nouchali* flower (NNF) was shown to reduce the adverse effects of t-BHP by attenuating oxidative DNA damage and reducing cellular ROS formulation. This process was accompanied by phosphorylation of MAP kinase (p38 kinase and ERK) as a downstream effect of upregulating Nrf2 nuclear translocation [81]. Polyphenol-rich *Nymphaea nouchali* leaf extract (NNLE) was also observed to act against OS by increasing the mRNA and protein level of antioxidant and phase II detoxifying enzymes, especially HO-1. NNLE supplement promoted Nrf2 translocation into the nucleus and the level of phosphorylated p38 kinase and ERK [82]. These results were consistent with other *in vitro* results reporting that quercetin [83], flavon-3-ols [84, 85], puerarin [86], and phenolic acids [87] activate HO-1 by suppressing the MAPK pathway to mediate antioxidant activity.

However, multiple potential mechanisms have been hypothesized for the separation of Keap1 and Nrf2 and Nrf2 nuclear translocation. According to the first mechanism, polyphenols were able to increase cellular Nrf2 levels. Alam et al. had shown that NNF extract supplement increased the protein level of Nrf2 in a time-dependent manner, reaching the maximum level at 12 h after treatment [81]. In addition, quercetin also suppressed the ubiquitination and proteasomal turnover of Nrf2 to maintain its stability [83]. Secondly, NNF extract supplement dose-dependently decreased the transcriptional and translational levels of Keap1 [81]. Quercetin was also found to significantly reduce posttranslational levels of Keap1 protein [83]. However, the main bioactive polyphenols, agrimonolide (AM) and desmethylagrimonolide (DM), have been proved to facilitate Keap1 degradation to further accelerate the release of Nrf2 [88].

### 3.3. Regulation of Oxidoreductase Enzyme System

#### 3.3.1. Induction of Antioxidant Enzymes

Cellular ROS levels are regulated by an enzymatic antioxidant system, such as SOD, catalase (CAT), and glutathione peroxidase (GSH-Px). SOD can transform superoxide into H_2_O_2_, and then, H_2_O_2_ is catalyzed by CAT and GSH-Px. The Nrf2-ARE pathway was found to activate the endogenous protective system, facilitating antioxidant capacity in cells [88]. Song et al. found that anthocyanin supplement upregulated the expressions of SOD and GSH-Px, and subsequently, attenuated lipid peroxidation in the serum, kidney, and liver in an aging mouse model [62]. Psotova et al. had detected the inhibitory ability of some flavonoids on lipid peroxidation. This study showed that the order of the inhibitory ability of flavonoids on lipid peroxidation was as follows: quercetin>baicalein>kaempferol>luteolin>apigenin [89]. RSV supplementation significantly restored the activities of SOD, GSH-Px, and CAT. When administered with resveratrol at the dose of 1 mg/kg, the activities of the above enzymes increased 1.4-, 1.5-, and 1.3-fold, respectively, compared to the control group [58]. Glutathione, as a major antioxidant, exists in almost every cell in reduced (GSH) and oxidized (GSSG) forms. The above enzymes play key protective functions against reactive species. Pereira et al. compared the structure-antioxidant activity of GSH combined with different kinds of flavonoids. All of tested flavonoids exhibited antioxidant properties; however, those carrying the catechol in B ring showed synergistic effects with GSH, except those with -OH group at C6, such as quercetin, (+)-catechin, fisetin, luteolin-7-O-glucoside, and taxifolin. In addition, adducts formed at C′2 and C′5 of the B ring exhibited a notably stronger antioxidant activity than those at C′6 and C′8 of the A ring [90].

#### 3.3.2. Inhibition of Oxidant Enzymes

Oxidant enzymes, a large collection of multiple enzymes, include nicotinamide adenine dinucleotide phosphate oxidase (NOX), xanthine oxidase (XOD), nitric oxide synthase (NOS), cyclooxygenase (COX), and lipoxygenase (LOX). They not only play essential roles in redox reactions but also promote cellular reactive oxygen and nitrogen (ROS/RNS) [91]. The NOX family members exhibit multiple cellular localizations, activated patterns, and types of produced ROS. However, as major producers of ROS, all those members could generate hydrogen peroxide and superoxide [92].

RSV has been found to attenuate particular matter-enhanced COX-2/prostaglandin E_2_ (PGE2) expression, NOX activity, and ROS generation by inhibiting the activation of oxidative stress signaling pathways (NF-*κ*B/NOX/ROS) in human fibroblast-like synoviocytes [51]. Nuclear factor *κ*B (NF-*κ*B) is a nuclear transcription factor participating in the regulation of various inflammation-related genes, which can be activated by several stimulations through different forms of radiation to oxidative stress. Resveratrol was shown to impair p65 NF-*κ*B translocation into the nucleus, and subsequently, inhibit binding with DNA in a dose-dependent manner [51, 58]. Resveratrol was confirmed to protect from oxidative stress by downregulation of protein kinase C-*α* (PKC-*α*) activation and NOX in A549 cell line and isolated human neutrophils [93, 94]. Gang et al. found that puerarin decreased NOX activity by suppressing activation of Ras-related C3 botulinum toxin substrate 1 (Rac1) and membrane translocation of oxidase subunits, and then, reduced ROS production [86]. RSV was shown to suppress NOX activity, including an O-methylation group, a 4′-OH group, and an extra -OH group. RSV was confirmed to inhibit 11*β*-hydroxysteroid dehydrogenase type 1 directly by decreasing cortisol production [95]. With supplementation of anthocyanins, both mRNA and protein level of XOD as well as XOD activity in the serum and liver were inhibited significantly in a dose-dependent manner [64]. Consistent with previous reports, polyphenol extracts of sweet cherry, containing hydroxycinnamic acids and flavonoids, were found to inhibit the XO system to reduce intracellular ROS [55]. Lin et al. compared the effects of a series of phenolic acids on XOD activity. A biochemical method was utilized to study the inhibitory ability of phenolic acids on XOD, and the order of inhibition was as follows: sinapic acid>ferulic acid>syringic acid>p*-*coumaric acid>chlorogenic acid>caffeic acid [96]. Based on previous studies, flavonoids inhibit XOD activity not only in a dose-dependent manner but also in a structure-activity manner. Hydroxyl attached to C′5 and C′7 and the double bond nitric oxide, as a common type of reactive nitrogen, participated in several physiological and pathological processes. RSV was found to be able to disrupt the formation of inducible nitric oxide synthase (iNOS) and nitric oxide (NO) production in macrophages [94, 97]. Another polyphenol, quercetin, was also shown to reduce the production of NO by downregulating the NF-*κ*B signaling pathway [98].

### 3.4. Restoration of Mitochondrial Function

Mitochondria, as one of the important organelles, are considered to play a vital role in energy production and the regulation of cellular homeostasis and energy production. However, extra ROS produced by impaired mitochondria, especially superoxide anion (O^2−^) and hydrogen peroxide (H_2_O_2_), contributes to a burden of oxidative stress. In turn, extra ROS leads to rapid depolarization of mitochondrial inner membrane potential and impairment of oxidative phosphorylation, further accelerating ROS generation and damage.

Cocultured with catechin-rich cocoa flavanol, both rat islets and pancreatic *β* cells have been demonstrated to exhibit enhanced insulin secretion and mitochondrial respiration. The activities of mitochondrial components, III, IV, and V, were also increased, along with induced production of adenosine triphosphate (ATP) [99]. Catechins were demonstrated to promote Nrf2 nuclear translocation, initiate the expression of related target genes, like nuclear respiratory factor 1 (Nrf1) and GA-binding protein transcription factor alpha subunit (GABPA), improve the cell's mitochondrial function, and reduce OS [99]. Using electron microscopy, (-)-epicatechin(EPI) was shown to alleviate the decline in quantity and volume densities of mitochondria induced by ischemia/reperfusion compared to sham-operated animals [100]. EPI was also proved to significantly increase the stability of mitochondrial membranes and defend calcium-triggered mitochondrial deformation and swelling by activating *δ*-opioid receptor (DOR) [101]. DOR was confirmed to activate extracellular signaling-regulated kinase (ERK) signaling and decrease the cytochrome c release, protecting neurons against oxidative damage [102]. At the microscopic level, the protection of EPI on the mitochondrial membrane was prominent when exhibiting less mitochondrial respiration suppression, lowering Ca^2+^ accumulation in mitochondria, along with a certain amount of NADPH [100]. Ca^2+^ has been confirmed to enhance the activity of the mitochondrial respiratory chain by activating a Ca^2+^-dependent dehydrogenase in the tricarboxylic acid (TCA) cycle, thus producing ATP by oxidative phosphorylation and stabilizing the mitochondrial membrane potential. However, upon excess accumulation of mitochondrial Ca^2+^, the permeability of the mitochondrial inner membrane increased significantly, impairing inner and outer membrane potential difference and mitochondrial respiration, eventually leading to failure of mitochondrial function [103].

RSV has been investigated in several studies, showing a significant protective effect on mitochondrial function. RSV was demonstrated to enhance the production of ATP and phosphocreatine in T2D female rat hearts in the reperfusion period. Biochemical analysis confirmed that RSV improved the levels of indicators of mitochondrial function, including an increase in total adenine nucleotide, creatine, and citrate synthase activity [104]. It should be noted that the effect of RSV on mitochondria depends very strictly on drug concentration. At low concentration (<50 *μ*M), RSV promoted cellular antioxidant ability, activating AMP-activated protein kinase- (AMPK-) and sirtuin 1- (SIRT1-) related signaling pathways to enhance the network formation of mitochondria against toxins and disease-related stress. However, when its concentration increased to more than 50 *μ*M, RSV destroyed cellular Ca^2+^ homeostasis, impaired mitochondrial membrane potential, and selectively activated caspases to induce cell death [103].

Taken together, this section provided an overview of these findings and is aimed at evaluating the potential medical feasibility of mitochondrial manipulation by polyphenols. We believe that the main pharmacological potential of polyphenols is closely associated with mitochondria, thus highlighting the advantage of these special cellular organelles as promising future therapeutic targets.

## 4. Challenges and Future Directions

This article reviews the classifications, chemical structures, sources, and potential roles of various kinds of polyphenols in metabolic disorders. Furthermore, acting against the oxidative stress is confirmed to be a major potential mechanism for the bioprotective effect of polyphenols in MetS. Polyphenols are naturally occurring drugs that rely on their various biological activities, not just antioxidant, and have gradually become an integral part of the dietary preventive treatment of many diseases, including MetS. However, various challenges still exist and are arduous. First, improving the bioavailability of polyphenols more effectively in order to promote their effectiveness is challenging. From several animal models and clinical trials, researchers have reached a consensus that polyphenols possess low bioavailability/high bioactivity paradox [3]. For example, the daily dosage of resveratrol in humans is much lower than that in mice (150 mg/day vs. 200-400 mg/kg/day) [105, 106]; however, the plasma resveratrol levels is about 2-23 folds higher in humans than in mice (231 ng/mL vs. 10-120 ng/mL) [106]. These differences may be attributed to different metabolic rates of resveratrol between humans and mice, suggesting that resveratrol may exert its effects at different concentration ranges in different mammals [107]. Due to these differences between humans and animals, we emphasize that clinical research is more desirable and informative. Second, a linear dose-response relationship existed in the polyphenol extract of *D*. *loddigesii* (DJP) and the improvement of diabetes [61], while a quadratic relationship was shown between flavonoid-rich cocoa (FRC) dose and flow-mediated vascular dilation (FMD) (*P*‐nonlinearity = 0.004), and its maximum effect was observed at 500 mg/d [37]. Moreover, a quadratic relationship was also observed between polyphenolic dose and HDLC levels (*P*‐nonlinearity = 0.06) [37]. Third, if the polyphenols are extracted as the medicine or as health supplements, attention should be paid to the activity loss and degradation of polyphenols during the extraction process. Fourth, the effects cannot be generalized for all kinds of polyphenols, because each polyphenol has its own unique features. In view of the fact that dietary polyphenols are a mixture, the research on potential mechanism is more complicated and difficult. Hence, it is difficult to compare doses achieved from polyphenolic supplements and polyphenol-rich food. The dose-response relationship between polyphenols and diseases is still largely unknown, requiring further studies to provide reasonable standards or critical doses for daily polyphenol intake.

Current lines of evidences suggest that polyphenols have the potential to alleviate phenotypes of MetS. However, further studies are required to identify the therapeutic potential of polyphenols in MetS progression. Safe doses also need to be determined, as the effects greatly vary among polyphenols and food sources, and no specific food or polyphenol is able to improve all phenotypes of MetS [17]. In addition, several undesirable properties of polyphenols have been disclosed, including genotoxicity [108], thyroid damage [109], hormone disorder [110], antinutritional activity [111], and interactions with other drugs [112]. Therefore, the potential cytotoxicity of polyphenols will become one of the important study directions in the future.

## Figures and Tables

**Table 1:** Summary of the classification, composition, and main sources of polyphenols.

Classifications	Subclasses	Compositions	Main sources
Flavonoids	Flavonols	Flavonols, kaempferol, quercetin, isorhamnetin, myricetin	Onions, shallots, spinach, green and black tea, dark chocolate, various fruits, vegetables, nuts
	Flavones	Apigenin, luteolin, tangeretin, nobiletin, wogonin, baicalein	Celery, parsley, some herbs, rooibos tea, citrus species, onion, garlic, pepper, Thai chili, citrus fruits, scutellaria, passiflora
	Isoflavones	Daidzein, genistein, glyciten	Leguminous plants, soybeans and soy products
	Flavanones	Naringenin, hesperetin, naringin, neohesperidin, rutinosides, narirutin, hesperidin	Flavedo of citrus fruits, bitter oranges, grapefruit, tomatoes, lemon, mandarin and grapefruit
	Anthocyanins	Pelargonidin, cyanidin, delphinidin, peonidin, petunidin, malvidin	Berries, cherries, red grapes, currants, red wines, oranges, the black varieties of soybeans, rice, beans, onions, potatoes, cabbage
	Flavan-3-ols	(+)-catechin, (−)-epicatechin, (+)-gallocatechin, (−)-epigallocatechin, (−)-epiafzelechin, (−)-epigallocatechin-3-O-gallate	Green tea, fruits, berries, cereals, nuts, chocolate, red wine
	Minor subclass of flavonoids	Chalcones	Tomatoes, licorice, shallots, and bean sprouts
		Dihydrochalcones (phloridzin, aspalathin, nothofagin)	Apples and apple products, rooibos tea
		Aurones	Vegetables and fruits
		Dihydroflavonols, flavan-3,4-diols	Biosynthetic intermediates of the flavonols and anthocyanins

Nonflavonoids	Phenolic acids	Hydroxybenzoic acids, gallic acid, protocatechuic acid	Gallotannins, raspberries, strawberries, blackberries, pomegranate, persimmon, walnuts, hazelnuts, grapes, wine, green and black teas, mangoes
	Hydroxycinnamic acids	Caffeic acid, ferulic acid, p-coumaric acid, sinapic acid	Flesh of grapes, blueberries, kiwis, plums, cherries, apples, coffee
	Stilbenes	Resveratrol	Red wines, grapes, peanuts, peanut products, plums, pine nuts
	Lignans	Syringaresinol, ecoisolariciresinol, matairesinol, medioresinol, pinoresinol, lariciresino	Linseed, algae, leguminous plants, cereals, vegetables, fruits
	Other polyphenols	Curcumin	Turmeric

**Table 2:** The antioxidant activity of polyphenols in both *in vitro* and *in vivo* models.

Authors, year	Sources	Main polyphenols	Cell models or animal models	Antioxidant activity changes
(Baird and Dinkova-Kostova 2011) [49]	L. coromandelica bark	Gallic acid, caffeic acid, (-)-epigallocatechin-3-gallate, chlorogenic acid, catechin	RAW 264.7 cells	Cellular ROS production↓, SOD, CSH-Px, and CAT activities↑
(Kim, Kim et al. 2011) [50]	Pycnogenol	Procyanidins, phenolic compounds	High glucose-treated renal tubular cells	Lipid peroxidation↓, total reactive species↓, superoxide↓, nitric oxide (NO(·))↓, peroxynitrite (ONOO(-))↓, iNOS↓, COX-2↓, NF-*κ*B nuclear translocation↓.
(Tsai, Hsu et al. 2017) [51]	Chemical hemisynthesis	Resveratrol	Human fibroblast-like synoviocytes	NADPH oxidase activity ↓, ROS generation↓
(Adefegha, Oyeleye et al. 2018) [52]	African crocus and wonderful kola seeds	Phenolic acids, flavonoids	Rat penile homogenate	FeSO_4_- and SNP-induced lipid peroxidation↓
(Lunder, Roskar et al. 2018) [53]	Coniferous	ND	Mouse C2C12 myoblast cells	Intracellular ROS production↓
(Oliveira, Dare et al. 2018) [54]	The leaves of Nectandra hihua	Flavonoids quercitrin, avicularin, juglalin, afzelin, astragalin	L929 fibroblasts	ROS production↓, lipid peroxidation inhibition↓
(Acero, Gradillas et al. 2019) [55]	Spanish local varieties of Prunus avium (L.)	ND	HepG2 cells	XOD↓, ROS↓
(Sun, Tao et al. 2019) [56]	Fresh citrus fruits	Flavonoids, phenolic acids	Intestinal HepG2 cells	CAA values ↑
(Nauman, Kale et al. 2018) [57]	Chemical hemisynthesis	Gallic acid, quercetin, rutin, acetylsalicylic acid	The ex-liver of mice	Peroxidative damage in microsomes↓, protein carbonyl in cytosolic fraction↓
(Liu, Ren et al. 2014) [58]	ND	Resveratrol	CK-exposed mice	The MDA activity↓, SOD, CSH-Px, and CAT activities↑
(Auberval, Dal et al. 2017) [59]	Red wine	Resveratrol	Wistar rats	Lipid peroxides↓, oxidative proteins↓.
(Jian, Ding et al. 2018) [60]	Loquat leaf	Flavonoids	PM_2.5_-induced NAFLD mice	Oxidative MDA↓, SOD↑
(Li, Chen et al. 2018) [61]	D. loddigesii, Dendrobium	Bibenzyls, phenanthrenes	Diabetic mice	The MDA activity↓, SOD, CSH-Px, and CAT activities↑
(Nauman, Kale et al. 2018) [57]	Chemical hemisynthesis	Gallic acid, acetylsalicylic acid	C57BL/6 mice	SOD, CSH-Px, and CAT activities↑, lipid peroxidation↓
(Song, Park et al. 2018) [62]	Walnut, chokeberry	Anthocyanins	Balb/c mice	MDA↓, lipid peroxidation↓, SOD and CSH-Px activities↑, antioxidant enzyme gene expression↑
(Zyzelewicz, Bojczuk et al. 2018) [63]	Cocoa bean	Flavan-3-ols, flavonols, phenolic acids	Male Wistar rats	GSH↑, GSSG↓
(Qian, Wang et al. 2019) [64]	Bilberry and black currant	Anthocyanins	Male ICR mice	SUA level↓, XOD activity↓, XOD mRNA and protein expressions↓

(↓): decrease; (↑): increase; ND: not detected; ROS: reactive oxygen species; NADPH: nicotinamide adenine dinucleotide phosphate; SNP: sodium nitroprusside; CAA: cellular antioxidant activity; iNOS: inducible nitric oxide synthase; COX-2: cyclooxygenase-2; NF-*κ*B: nuclear factor-kappa; XOD: xanthine oxidase; SOD: superoxide dismutase; CSH-Px: glutathione peroxidase; GSH: glutathione; GSSG: oxidized glutathione; CK: cigarette smoke; MDA: malondialdehyde; CAT: chloramphenicol acetyl transferase; SUA: serum uric acid; NAFLD: nonalcoholic fatty liver disease.

**Table 3:** Summary of results from clinical trials of polyphenols in individuals with MetS.

Authors, year	Sources	Main polyphenols	Individuals	Duration	Bioactivities	Improvements
(Faghihzadeh, Adibi et al. 2015) [15]	Resveratrol capsules	Resveratrol	50 NAFLD patients	12 weeks	ND	ALT↓, hepatic steatosis↓
(Costabile, Vitale et al. 2018) [21]	Red grape pomace	Anthocyanins, flavan-3-ol, procyanidins	12 healthy men	1 week	ND	Postprandial insulin incremental area↓, insulin secretion index↓, IS index↑
(Fang, Kim et al. 2018) [22]	Mango	Gallic acid, gallotannin, galloylglycosides, flavonoids	12 lean and 9 obese participants	6 weeks	Anti-inflammation: IL-8↓ and MCP-1 ↓.	In lean participants: systolic BP↓ In obese participants: HbA1c and PAI-1↓
(Dallas, Gerbi et al. 2014) [27]	Sinetrol-XPur	Catechin, naringin	95 overweight subjects	12 weeks	Antioxidation: MDA↓, SOD↑, GSH↑ Anti-inflammation: CRP↓, fibrinogen↓	Waist and hip circumference↓, abdominal fat↓, body weight↓
(Astell, Mathai et al. 2013) [28]	C. fimbriata extract	Gallic acid	43 overweight and obese subjects	12 weeks	ND	Waist circumference↓, WHR↓ palatability of the test meal↓, sodium intake↓, body weight↓, BMI↓, hip circumference↓, systolic BP↓, HR↓, TG↓, total fat and saturated fat intake↓
(Taubert, Roesen et al. 2007) [36]	Cocoa-containing foods	Ericatechin, procyanidin dimer, flavonols	44 untreated upper-range prehypertension or stage 1 hypertension	18 weeks	Antioxidation: fasting plasma levels of S-nitrosoglutathione↑	Systolic BP↓, diastolic BP↓, hypertension prevalence↓
(Rangel-Huerta, Aguilera et al. 2015) [38]	Orange juice	Anthocyanins	100 obese or overweight adults	12 weeks	Antioxidation: 8-OHdG↓, GR↓, erythrocyte catalase↓, 8-isoPGF2*α*↓, SOD↑	Body mass index↓, waist circumference↓, leptin↓, systolic and diastolic BP↓
(Chai, Davis et al. 2018) [39]	Tart cherry juice	Gallic acid	17 men and 20 women	12 weeks	ND	Systolic BP↓, LDLC↓, TC↓
(Erlund, Koli et al. 2008) [41]	Berry	Quercetin, caffeic acid, vanillic acid,	72 unmedicated subjects with cardiovascular risks	8 weeks	ND	HDLC↑, systolic BP↓
(Samavat, Newman et al. 2016) [42]	Green tea	Catechins	936 women	12 months	ND	Circulating TC↓, LDLC↓ Non-HDLC↓.
(Faghihzadeh, Adibi et al. 2014) [46]	Resveratrol capsules	Resveratrol	50 NAFLD patients	12 weeks	Anti-inflammation: IL-6↓, NF-*κ*B activity↓, serum CK-18 ↓	Weight↓, body mass index↓, waist circumference↓, ALT↓, hepatic steatosis grade↓
(Chen, Zhao et al. 2015) [47]	Resveratrol capsules	Resveratrol	60 NAFLD patients	3 months	Anti-inflammation: NF-*κ*B activity ↓, serum CK-18 ↓	AST↓, glucose↓, LDLC↓, ALT↓, TC↓HOMA-IR↓TNF-*α*↓, CK-18 fragment↓, FGF21↓, adiponectin level↑
(Martinez-Maqueda, Zapatera et al. 2018) [65]	Dried grape pomace	Anthocyanins, flavan-3-ol	50 participants with at least one phenotype of MetS	6 weeks	ND	IR↓, fasting insulinemia↓, Is↑
(Tynkkynen, Mursu et al. 2012) [66]	Chocolate	Epicatechin	45 nonsmoking volunteers	3 weeks	Antioxidation: oxidation susceptibility of serum lipids↓	HDL↑
(Mursu, Voutilainen et al. 2005) [67]	Polyphenol-rich phloem	Catechins	75 nonsmoking hypercholesterolemic men	4 weeks	Antioxidation: Oxidation resistance of total serum lipids↑	Lipid peroxidation↓
(Espinosa-Moncada, Marin-Echeverri et al. 2018) [68]	Vaccinium meridionale Swartz (agraz)	Anthocyanins	40 women with MetS	4 weeks	Antioxidation: serum antioxidant capacity↑, urinary 8-OHdG ↓ Anti-inflammation: Hs-CRP levels ↓	Serum antioxidant capacity↑, DNA oxidative damage↓
(Vetrani, Vitale et al. 2018) [69]	ND	Flavonoids, flavan-3-ols, phenolic acids, flavonols	78 individuals with at least one features of the MetS	8 weeks	Antioxidation: urinary isoprostanes↓	Postprandial lipid response↓, VLDL↓, early insulin secretion↑
(Chiva-Blanch, Urpi-Sarda et al. 2013) [70]	Red wine	Catechin, epicatechin, malvidin-3-glucoside, gallic acid	73 male moderate alcohol consumers	4 weeks	ND	Plasma insulin↓, HOMA-IR↓, HDLC↑, Apo A-I↑, Apo A-II↑, lipoprotein↓

(↓): decrease; (↑): increase; ND: not detected. MetS: metabolic syndrome; IR: insulin resistance; IS: insulin sensitivity; IL: interleukin; MCP-1: monocyte chemotactic protein-1; HbA1c: glycosylated hemoglobin; PAI-1: plasminogen activator inhibitor 1; BP: blood pressure; MDA: malondialdehyde; SOD: superoxide dismutase; GSH: glutathione; CRP: C-reactive protein; WHR: waist to hip ratio; BMI: body mass index; HR: heart rate; TG: triacylglycerol; LDLC: low-density lipoprotein cholesterol; 8-OHdG: 8-hydroxydeoxyguanosine; GR: glutathione reductase; HDLC: high-density lipoprotein cholesterol; TC: total cholesterol; VLDL: very low-density lipoprotein; NAFLD: nonalcoholic fatty liver disease; ALT: alanine aminotransferase; NF-*κ*B: nuclear factor-kappa B; CK: cytokeratin; FGF: fibroblast growth factor; 8-isoPGF2*α*: 8-iso-prostaglandin F2*α*; AST: aspartate aminotransferase; HOMA-IR: homeostasis model assessment insulin resistance index; TNF-*α*: tumor necrosis factor-alpha; hs-CRP: high-sensitivity C-reactive protein; Apo: apolipoprotein.
